# PLG基因复合杂合突变致遗传性纤溶酶原缺陷症：一项家系研究与机制分析

**DOI:** 10.3760/cma.j.cn121090-20250317-00137

**Published:** 2026-01

**Authors:** 一凡 芦, 丹丹 虞, 琦煜 徐, 凤娇 王, 朗译 秦, 明山 王, 丽红 杨

**Affiliations:** 温州医科大学附属第一医院医学检验中心，浙江省检验诊断及转化研究重点实验室，温州 325015 Department of Clinical Laboratory, Key Laboratory of Clinical Laboratory Diagnosis and Translational Research of Zhejiang Province, the First Affiliated Hospital of Wenzhou Medical University, Wenzhou 325015, China

**Keywords:** 纤溶酶原, 遗传性纤溶酶原缺陷症, 体外表达, 血栓形成, Plasminogen, Hereditary plasminogen deficiency, In vitro expression, Thrombus formation

## Abstract

**目的:**

对1例遗传性纤溶酶原（PLG）缺陷症患者所携带的复合杂合突变进行分子机制研究。

**方法:**

先证者因“左侧肢体乏力2 d”至温州医科大学附属第一医院就诊。对先证者及其家系成员（3代共8人）采用发色底物法和酶联免疫吸附试验（ELISA）分别检测PLG活性（PLG∶A）和PLG抗原（PLG∶Ag）含量。采用DNA直接测序法明确基因突变位点。利用生物信息学软件分析突变位点的保守性和致病性。构建突变蛋白模型观察突变前后蛋白结构的变化。构建重组质粒表达载体，并采用实时荧光定量PCR法（qRT-PCR）、ELISA、Western blot实验对重组PLG蛋白进行体外表达研究。

**结果:**

先证者PLG∶A为27％（参考范围：80％～120％），PLG∶Ag为103％（参考范围：50％～150％），属于Ⅱ型PLG缺陷症。基因分析显示，先证者第14和15外显子分别存在c.1702G>A（p.Gly568Arg）和c.1858G>A（p.Ala620Thr）复合杂合错义突变。c.1702G>A位点在7种同源物种中均为高度保守，且生物信息学软件预测其具有致病性。蛋白模型分析显示p.Gly568Arg突变导致氨基酸侧链延长并与p.Leu686之间形成新的氢键。体外表达显示2个突变均未导致基因的转录、蛋白质合成和分泌异常，但在培养上清液中两个突变的PLG∶A/PLG∶Ag比值均较野生型明显降低（Ala620Thr：0.598±0.114对1.000，*P*＝0.013；Gly568Arg：0.412±0.079对1.000，*P*＝0.022）。

**结论:**

p.Gly568Arg和p.Ala620Thr杂合错义突变与该家系先证者PLG∶A降低有关，该2个突变可能通过改变蛋白质的空间构象导致其功能异常。

## Abstract (en)

**Objective:**

To investigate the molecular mechanisms underlying compound heterozygous mutations in a patient with hereditary plasminogen（PLG）deficiency.

**Methods:**

The proband presented to the First Affiliated Hospital of Wenzhou Medical University with a 2-day history of left-sided limb weakness. Plasminogen activity（PLG∶A）and plasminogen antigen（PLG∶Ag）were measured by chromogenic substrate and enzyme‑linked immunosorbent assays, respectively, in the proband and family members（eight individuals across three generations）. Sanger sequencing was performed to identify the PLG mutation sites. Bioinformatic analyses were conducted to assess evolutionary conservation and to predict pathogenicity of the mutation sites. Mutant protein models were constructed to examine mutation-induced structural changes. Recombinant plasmid expression vectors were constructed, and in vitro expression of recombinant PLG protein was studied using quantitative real-time PCR（qRT-PCR）, ELISA, and Western blot analysis.

**Results:**

The proband's PLG∶A was 27％（reference range, 80％–120％）and PLG∶Ag was 103％（reference range, 50％–150％）, consistent with type Ⅱ plasminogen deficiency. Genetic analysis revealed compound heterozygous missense mutations in the proband: c.1702G>A（p. Gly568Arg）in exon 14 and c.1858G>A（p. Ala620Thr）in exon 15. The c.1702G>A site is highly conserved across seven species, and is predicted to be pathogenic by bioinformatic tools. Protein modeling showed that p. Gly568Arg introduces a longer side chain and forms a new hydrogen bond with Leu686. In vitro expression showed that neither mutation caused abnormalities in PLG transcript levels, protein expression, or secretion; however, the PLG∶A/PLG∶Ag ratios in the culture supernatants were significantly lower than wild-type for both variants（Ala620Thr: 0.598 ± 0.114 *vs* 1.000, *P*＝0.013; Gly568Arg: 0.412 ± 0.079 *vs* 1.000, *P*＝0.022）.

**Conclusion:**

The heterozygous missense mutations p.Gly568Arg and p. Ala620Thr are associated with decreased PLG∶A in the family proband and may cause functional impairment by altering protein conformation.

纤溶酶原（PLG）基因定位于人类染色体6q26～27区域，包含19个外显子和18个内含子，基因组全长52.5 kb，其cDNA长度为2.7 kb，编码由810个氨基酸残基组成的单链糖蛋白，相对分子质量约为90×10^3^［1]。PLG蛋白具有特征性的结构域，包括N端多肽（NTP）、5个高度同源的kringle结构域及C端丝氨酸蛋白酶结构域[2]。在血浆中，PLG主要以两种形式存在：谷氨酸-PLG和赖氨酸-PLG[3]，这两种形式在肝脏和角膜中合成，维持纤溶系统平衡。遗传性PLG缺陷症是一种极其罕见的血栓性疾病，其患病率约为1.6/100万[4]。在临床工作中观察发现，部分脑梗死患者伴有PLG活性（PLG∶A）降低，提示PLG功能异常可能与脑血管疾病的发生相关。本研究通过对1例PLG∶A降低的脑梗死患者及其家系成员进行基因检测和体外表达研究，旨在阐明遗传性PLG缺陷症分子致病机制，为PLG缺陷相关疾病的诊断和治疗提供理论依据。

## 对象与方法

1. 家系资料：先证者，女，60岁。2024年7月因“左侧肢体无力2 d”至温州医科大学附属第一医院就诊，根据磁共振成像检查诊断为“脑梗死”。实验室检查发现PLG∶A降至27％（参考范围：75％～140％），PLG抗原（PLG∶Ag）为103％（参考范围：50％～150％），其他凝血指标均无明显异常，肝肾功能正常。家系调查发现除先证者外其余家系成员平素均无血栓形成相关临床表现，家系图见图1。本研究通过本院伦理委员会审查（伦审KY2022-R193），且所有参与者均签署知情同意书。

**图1:**
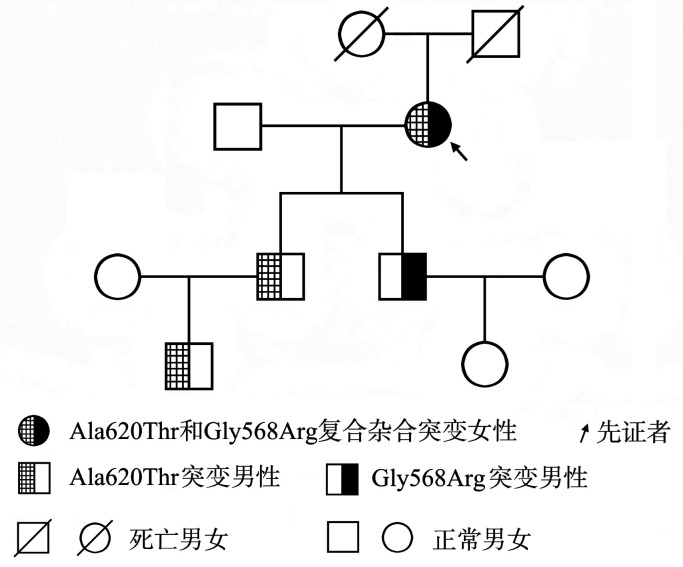
遗传性纤溶酶原缺陷症家系图

2. 标本采集与处理：采集所有参与者的外周静脉血样本，用0.109 mol/L枸橼酸钠抗凝。在室温条件下1 500×*g*离心15 min，乏血小板血浆（PPP）用于凝血功能检测，血细胞用于提取基因组DNA。

3. 凝血指标检测：在Stago-STA-R-Max全自动凝血仪（法国Diagnostica Stago公司产品）上采用发色底物法检测PLG∶A、蛋白C活性（PC∶A）、抗凝血酶活性（AT∶A）；凝固法检测蛋白S活性（PS∶A）。用酶联免疫吸附法测定PLG∶Ag。以上所有操作严格按照试剂说明书进行。

4. DNA提取及PCR扩增：使用DNA提取试剂盒（北京天根生化科技有限公司产品）提取外周血基因组DNA。参考文献[5]设计PLG基因外显子及其侧翼序列共19对引物。PCR扩增产物测序和引物合成均由上海赛恒生物科技有限公司完成。

5. DNA测序分析：采用DNA直接测序法对PLG基因（Gene ID：5340）进行双向测序。使用Chromas软件对测序结果进行分析，并与NCBI（http://www.ncbi.nlm.nih.gov/homologene）公布的PLG基因序列（ID：5340）进行比对分析。检出先证者的潜在致病突变位点，进一步在家系成员中进行靶向测序验证。

6. 生物信息学预测及蛋白模型构建：p.Ala620Thr保守性分析、致病性分析及结构变化既往已报道，见参考文献[4]，由于在基因转录过程中会剪切掉包含19个氨基酸的前端内含子序列，进而可翻译为成熟的蛋白质，故该位点也可表示为p.Ala601Thr，在本文中均为未剪切的氨基酸位点表述方式。采用MutationTaster、SIFT、PROVEAN和FATHMM 4个软件预测p.Gly568Arg突变是否具有致病性；用ClustalⅩ-2.l-win软件将人类与美国NCBI基因库中提供的其他6种同源物种进行多序列比对，评估p.Gly568Arg突变位点的保守性；用PyMol软件（Python-2.7）构建p.Gly568Arg突变位点相应蛋白模型，分析突变前后的蛋白质空间结构变化。

7. 构建野生型和突变型PLG表达载体：以pCDH-cop GFP-T2A-Puro质粒（购自南京擎科生物技术有限公司）为模板，构建野生型PLG重组蛋白表达载体（PLG-WT）。用Primer X在线引物设计软件（http://www.bioinformatics.org/primerx/）设计特异性突变引物，并参照QuikChange Lightning Site-Directed Mutagenesis Kit（美国Stratagene公司产品）操作手册，在PLG-WT基础上进行定点突变。为确保突变质粒构建的准确性，将所得突变质粒送至南京擎科生物技术有限公司进行测序验证，经序列比对确认，成功构建目标突变质粒。

8. 细胞培养和转染：采用标准细胞复苏方法，将冻存于−80 °C的HEK293T细胞（杭州杰西摩生物科技有限公司产品）迅速解冻，接种于含10％胎牛血清的DMEM完全培养基中，置于37 °C、5％ CO_2_恒温培养箱中进行复苏培养。待细胞生长24 h后，当细胞融合度达到70％～80％时，选取生长状态良好的细胞进行细胞转染实验。使用Lipofect5000转染试剂（常州百代生物科技股份有限公司产品）分别将构建好的野生型、突变型质粒转染至生长状态良好的HEK293T细胞中，以进行后续蛋白表达分析。

9. PLG基因转录水平的检测：瞬时转染48 h后，采用RNAiso Plus试剂（日本TaKaRa公司产品）提取转染细胞内总RNA，使用HiScriptⅡQ RT SuperMix for qPCR试剂（南京诺唯赞生物科技股份有限公司产品）进行逆转录合成cDNA。根据Taq Pro Universal SYBR qPCR Master Mix试剂（南京诺唯赞生物科技股份有限公司产品）说明书，运用实时荧光定量PCR（qRT-PCR）技术检测野生型和突变型PLG基因mRNA表达水平。采用Applied Biosystems™ QuantStudio 5 qPCR System软件分析PLG基因和GAPDH基因的Ct值，以GAPDH为内参，通过2^−ΔΔCt^法计算野生型和突变型PLG基因mRNA的相对表达量。每种重组蛋白设定3个重复孔，至少进行3次独立实验。数据以均数±标准差表示。PLG及GAPDH基因特异性引物序列详见表1。

**表1:** PLG和GAPDH基因引物序列

基因名称	正向序列	反向序列
PLG	5′-GCGACATTCTTGAGTGTGAA-3′	5′-GGGGTTACGACAGTAATTCT-3′
GAPDH	5′-AGAAGGCTGGGGCTCATTTG-3′	5′-AGGGGCCATCCACAGTCTTC-3′

10. PLG蛋白表达水平的检测：转染48 h后，分别收集转染阳性细胞及其培养上清。收集经含PMSF的RIPA缓冲液裂解后细胞，培养上清液则通过超滤管浓缩处理。提取蛋白并变性后采用10％ SDS-PAGE分离蛋白样品，转膜后依次进行封闭、一抗（PLG和GAPDH兔抗人IgG单克隆抗体，美国Proteintech公司产品）和二抗（辣根过氧化物酶标记山羊抗兔IgG，上海碧云天公司产品）孵育。使用ECL超敏化学发光检测试剂盒（上海雅酶公司产品）显影分析。同时，采用ELISA试剂盒（上海江莱生物有限公司产品）定量检测细胞裂解液和培养上清中的PLG∶Ag水平，并在Stago-STA-R-Max全自动凝血仪上采用发色底物法检测培养上清中的PLG∶A，以PLG∶A/PLG∶Ag比值表示特异活性，并将PLG-野生型的特异活性设定为100％作为参照。

## 结果

1. 凝血功能检查结果：先证者的PLG∶A显著降低，仅为27％，而其PLG∶Ag未降低，为103％；家系成员中，先证者的两个儿子及孙子PLG∶A均明显降低，而PLG∶Ag水平均处于正常范围。家系其他成员的凝血指标均在正常范围内，详见表2。

**表2:** 先证者及其家系成员基因型检测结果及临床表现

家系成员	PLG∶A（％）	PLG∶Ag（％）	PC∶A（％）	PS∶A（％）	AT∶A（％）	突变类型	临床表现
先证者（I_1_）	27	103	76	77	99	p.Ala620Thr、p.Gly568Arg	脑梗死
丈夫（I_2_）	93	94	81	69	103	野生型	无
大儿媳（Ⅱ_1_）	137	75	73	87	98	野生型	无
大儿子（Ⅱ_2_）	54	126	83	84	100	p.Ala620Thr	无
小儿子（Ⅱ_3_）	42	104	77	73	105	p.Gly568Arg	无
小儿媳（Ⅱ_4_）	110	87	70	82	102	野生型	无
孙子（Ⅲ_1_）	55	119	75	67	99	p.Ala620Thr	无
孙女（Ⅲ_2_）	131	107	72	66	104	野生型	无

参考范围	75～140	50～150	70～130	65～135	96～118		

**注** PLG∶A：纤溶酶原活性；PLG∶Ag：纤溶酶原抗原；PC∶A：蛋白C活性；PS∶A：蛋白S活性；AT∶A：抗凝血酶活性

2. 基因测序结果分析：先证者PLG基因第14号外显子存在c.1702G>A杂合错义突变，导致p.Gly568Arg；第15号外显子存在c.1858G>A杂合错义突变，导致p.Ala620Thr。先证者的两个儿子分别携带单一杂合突变（p.Ala620Thr或p.Gly568Arg），孙子为p.Ala620Thr突变携带者，其余家系成员均为野生型。具体基因型分布详见表2和图2。正常人群中未检测到c.1702G>A突变，排除了其为多态性的可能性。

**图2:**
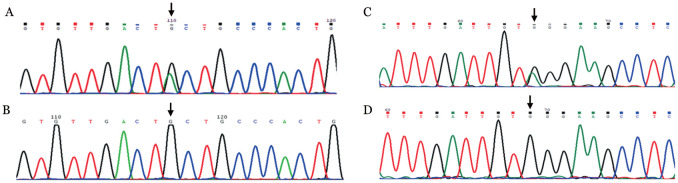
PLG基因外显子测序结果（箭头所示为突变位点） **A** 先证者c.1858G>A杂合突变型；**B** 先证者丈夫c.1858G>A野生型；**C** 先证者c.1702G>A杂合突变型；**D** 先证者丈夫c.1702G>A野生型

3. 生物信息学分析：四个生物信息学软件显示p.Gly568Arg突变具有致病性（计算分数：SIFT为0.002、FATHMM为−0.185、MutationTaster为1.000和PROVEAN为−5.85）。通过ClustalⅩ-2.l-win软件进行同源物种序列比对，发现p.Gly568Arg在7个同源物种中呈现完全保守（图3）。蛋白三维结构模型分析发现，p.Gly568Arg突变后，氨基酸侧链延长，同时与p.Leu686之间新增了一个氢键（图4）。

**图3:**
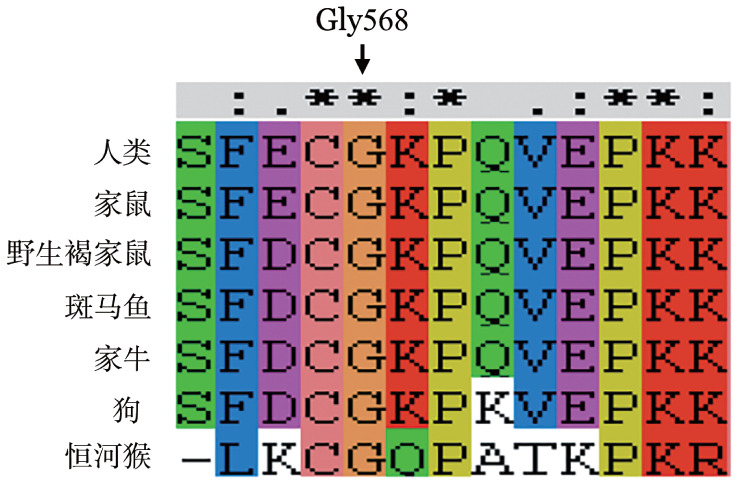
p.Gly568Arg不同物种间保守性分析

**图4:**
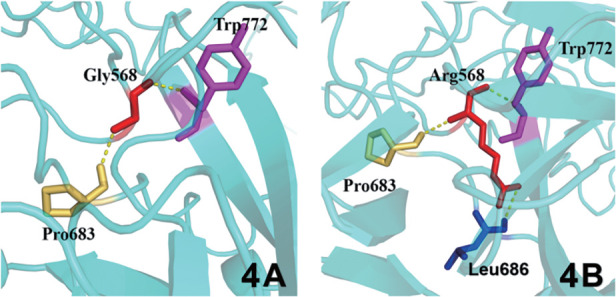
p.Gly568Arg蛋白模型结构图 **A** 突变前；**B** 突变后

4. 重组蛋白体外表达研究：与PLG-野生型相比，PLG-p.Ala620Thr（*P*＝0.834）和PLG-p.Gly568Arg（*P*＝0.786）突变型PLG基因mRNA差异均无统计学意义。Western blot实验结果表明细胞裂解液和培养上清液中PLG-p.Ala620Thr和PLG-p.Gly568Arg突变型蛋白差异均无统计学意义（均*P*>0.05）。ELISA结果显示，PLG-p.Ala620Thr和PLG-p.Gly568Arg的PLG∶Ag含量，在细胞裂解液中分别为（88.7±2.6）％、（93.2±1.8）％，在培养上清液中分别为（98.7±4.9）％、（101.2±5.1）％。两种突变的细胞裂解液和培养上清液中的PLG∶Ag含量与PLG-野生型相比差异均无统计学意义（均*P*>0.05），但细胞上清液的PLG∶A/PLG∶Ag比值均较PLG-野生型显著降低（PLG-p.Ala620Thr：0.598±0.114对1.000，*P*＝0.013；PLG-p.Gly568Arg：0.412±0.079对1.000，*P*＝0.022）（图5）。

**图5:**
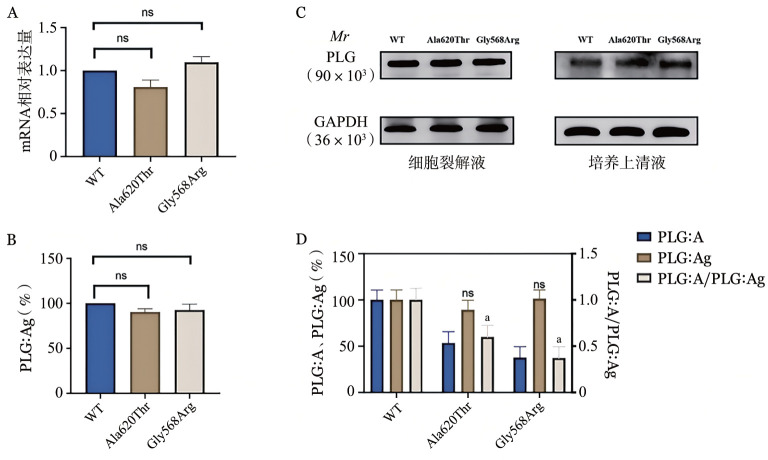
纤溶酶原（PLG）重组蛋白转录组及蛋白表达水平分析（^ns^*P*>0.05、^a^*P*<0.001） **A** PLG野生型（WT）和突变型mRNA相对表达水平；**B** 细胞裂解液PLG抗原（PLG∶Ag）水平；**C** 细胞裂解液和培养上清液中的PLG蛋白表达水平；**D** 细胞上清液PLG活性（PLG∶A）、PLG∶Ag水平及比值

## 讨论

遗传性PLG缺陷症是一种常染色体隐性遗传病，主要由PLG基因突变导致PLG功能异常[5]。根据PLG∶A和PLG∶Ag水平的变化可分为两种临床类型:Ⅰ型缺陷为低PLG血症，患者血浆中PLG∶A和PLG∶Ag均显著降低，特征性临床表现为结膜、齿龈等黏膜表面反复形成木质样假膜，严重者可导致视力障碍[2]；Ⅱ型缺陷为异常PLG血症，患者血浆中PLG∶A降低而PLG∶Ag正常，主要临床表现为反复静脉血栓栓塞事件，特别是当患者合并获得性血栓形成危险因素（如手术、创伤、妊娠等）时，血栓形成风险显著增加[6]。

本研究报道的先证者为脑梗死患者，存在多次脑梗死的发病史，本次入院后使用阿司匹林抗血小板、重组组织型PLG激活剂（rt-PA）溶栓及低分子肝素抗凝等药物治疗后病情明显好转。实验室检查发现，该先证者PLG∶A明显降低（仅27％）而PLG∶Ag正常，其他凝血指标无明显异常。基因检测发现该先证者PLG基因上存在两个错义突变，分别为c.1702G>A（p.Gly568Arg）和c.1858G>A（p.Ala620Thr），先证者的两个儿子分别为p.Ala620Thr和p.Gly568Arg的单杂合子携带者，孙子为p.Ala620Thr单杂合子携带者，其余家系成员为野生型。据美国医学遗传学与基因组学学会（ACMG）指南[7]对突变进行致病性评估，p.Gly568Arg和p.Ala620Thr突变满足致病性证据（PS1、PM2、PP3、PP4）。结合实验室检查结果和临床表现，先证者被确诊为Ⅱ型遗传性PLG缺陷症（异常PLG血症）。

p.Ala620Thr突变首先由Ichinose等[8]在1991年报道，而p.Gly568Arg突变在国内外文献中均未见报道，属于国际首次发现的新突变位点。通过多重序列比对分析发现，p.Ala620[4]和p.Gly568位点在多个同源物种间呈现完全保守，提示这两个位点在PLG蛋白结构和功能中具有重要功能。进一步的蛋白结构模型分析发现，p.Ala620位于PLG蛋白催化活性中心附近，其突变后的p.Thr620与催化三联体（His603-Asp646-Ser741）之间形成3个新的氢键[9]，这种空间结构的改变可能导致催化中心构象异常，从而影响PLG的酶活性。p.Gly568邻近Arg561-Val562，该位点是rt-PA或尿激酶型PLG激活剂（u-PA）介导的PLG激活的关键位点[10]。蛋白模型显示，p.Gly568Arg突变导致p.Arg侧链延长，并在p.Arg末端与p.Leu686形成新的氢键连接，可能干扰t-PA或u-PA对Arg561-Val562肽键的正常切割，从而阻碍PLG向纤溶酶的转化。纤溶酶生成减少或功能下降，会导致纤维蛋白清除能力减弱，易形成血栓。复合杂合突变常有协同效应，致病性通常强于单杂合突变，易导致更严重的功能缺陷。本研究中存在复合杂合突变的先证者PLG∶A明显低于携带单杂合突变的家系成员，导致先证者血栓形成风险增加，其发生脑梗死可能与PLG∶A明显降低有关。

为了进一步确定突变导致PLG∶A降低的致病性机制，本研究构建了PLG-野生型、PLG-p.Ala620Thr和PLG-p.Gly568Arg的质粒载体，并将其转染至HEK293T细胞中进行体外表达研究。通过qRT-PCR实验发现转染后细胞内mRNA转录与野生型相比差异无统计学意义，说明两个突变均未影响PLG基因的转录。通过Western blot实验进一步证实，PLG-p.Ala620Thr和PLG-p.Gly568Arg突变型蛋白在细胞裂解液和培养上清液中的表达水平与PLG-野生型相比差异无统计学意义，提示突变未影响PLG蛋白的合成和分泌过程。ELISA检测结果与Western blot结果基本一致，但在培养上清液中2个突变体的PLG∶A/PLG∶Ag比值均较PLG-野生型显著降低，提示突变可能通过改变蛋白质的空间构象导致异常PLG生成，进而影响其功能活性，最终导致Ⅱ型PLG缺陷症。遗传性PLG缺陷症是一种罕见疾病，目前缺乏精准的诊断标准和有效的治疗方案。临床上主要依赖输注新鲜冷冻血浆进行治疗，而我国尚未批准PLG替代治疗药物上市[11]。

综上所述，本研究通过对1例PLG∶A降低的脑梗死患者进行表型、基因型分析及体外表达研究，发现p.Ala620Thr和p.Gly568Arg复合杂合突变是引起该患者PLG∶A降低导致血栓发生的原因之一，其中p.Gly568Arg为国内外首次报道。p.Ala620Thr和p.Gly568Arg导致蛋白构象异常而影响其功能活性。
